# Perioperative hemodynamics and risk for delirium and new onset dementia in hip fracture patients; A prospective follow-up study

**DOI:** 10.1371/journal.pone.0180641

**Published:** 2017-07-10

**Authors:** Bjørn Erik Neerland, Maria Krogseth, Vibeke Juliebø, Anette Hylen Ranhoff, Knut Engedal, Frede Frihagen, Johan Ræder, Torgeir Bruun Wyller, Leiv Otto Watne

**Affiliations:** 1 Oslo Delirium Research Group, Department of Geriatric Medicine, Oslo University Hospital, Oslo, Norway; 2 Institute of Clinical Medicine, University of Oslo, Oslo, Norway; 3 Old Age Psychiatry Research Network, Telemark Hospital Trust and Vestfold Hospital Trust, Tønsberg, Norway; 4 Faculty of Health Science, University College of Southeast Norway, Drammen, Norway; 5 Diakonhjemmet Hospital, Oslo, Norway; 6 Department of Clinical Science, University of Bergen, Bergen, Norway; 7 Norwegian National Advisory Unit on Ageing and Health, Vestfold Health Trust, Tønsberg, Norway; 8 Department of Orthopaedic Surgery, Oslo University Hospital, Oslo, Norway; 9 Department of Anaesthesiology, Oslo University Hospital, Oslo, Norway; 10 Institute of Basic Medical Sciences, University of Oslo, Oslo, Norway; Cardiff University, UNITED KINGDOM

## Abstract

**Background:**

Delirium is common in hip fracture patients and many risk factors have been identified. Controversy exists regarding the possible impact of intraoperative control of blood pressure upon acute (delirium) and long term (dementia) cognitive decline. We explored possible associations between perioperative hemodynamic changes, use of vasopressor drugs, risk of delirium and risk of new-onset dementia.

**Methods:**

Prospective follow-up study of 696 hip fracture patients, assessed for delirium pre- and postoperatively, using the Confusion Assessment Method. Pre-fracture cognitive function was assessed using the Informant Questionnaire of Cognitive Decline in the Elderly and by consensus diagnosis. The presence of new-onset dementia was determined at follow-up evaluation at six or twelve months after surgery. Blood pressure was recorded at admission, perioperatively and postoperatively.

**Results:**

Preoperative delirium was present in 149 of 536 (28%) assessable patients, and 124 of 387 (32%) developed delirium postoperatively (incident delirium). The following risk factors for incident delirium in patients without pre-fracture cognitive impairment were identified: low body mass index, low level of functioning, severity of physical illness, and receipt of ≥ 2 blood transfusions. New-onset dementia was diagnosed at follow-up in 26 of 213 (12%) patients, associated with severity of physical illness, delirium, receipt of vasopressor drugs perioperatively and high mean arterial pressure postoperatively.

**Conclusion:**

Risk factors for incident delirium seem to differ according to pre-fracture cognitive status. The use of vasopressors during surgery and/or postoperative hypertension is associated with new-onset dementia after hip fracture.

## Introduction

A hip fracture is a potentially devastating event, and serious surgical and medical complications occur frequently [1]. One-year mortality is 25% and about 25% of patients who were home dwelling before the fracture will need a permanent nursing home placement afterwards [2]. The median age of hip fracture patients is above 80 years [3].

Delirium, characterised by an acute disturbance in awareness, attention and cognitive function [4] is one of the most common complications in hip fracture patients. Delirium has a fluctuating course and is usually reversible. However, evidence suggests an association between delirium and future cognitive decline and development of dementia during the months after the delirium episode [5–7]. Dementia is a chronic syndrome characterized by cognitive decline, impairment in activities of daily living and a change in social abilities and behaviour[8]. The term "cognitive impairment" has no universal definition, but will in this paper denote a condition in which a cognitive decline has occurred, but in which it is uncertain or cannot be firmly established whether the formal diagnostic criteria for dementia are fulfilled.

Several established risk factors are identified for preoperative and postoperative delirium, including age, comorbidities and pre-fracture dementia [9]. There is some controversy about the cognitive consequences of intraoperative control of blood pressure. Some studies have found that intraoperative hypotension is a predictor of postoperative delirium [10–13], whereas others have not found such an association [14–17]. Previous studies in hip fracture patients (n = 3) are rather small (n about 100) and have conflicting results[10, 11, 18]. Most studies on intraoperative hypotension examine patients undergoing elective surgery, and several have excluded patients with preoperative dementia. Pre-fracture dementia is the most consistently reported predisposing factor for delirium, and is common among hip fracture patients [19]. Thus, patients with dementia should be included to increase the external validity of the findings.

There are some data suggesting that use of vasopressors (in particular phenylephrine) is associated with lower cerebral oxygen saturation [20] which in turn is thought to cause cognitive problems in the postoperative phase [21]. Little is known about potential short-term and long-term cognitive outcomes of vasopressor use.

The main objective of this study was to study risk factors for preoperative delirium, for incident delirium, and for new-onset dementia in the months after surgery in hip fracture patients, with emphasis on perioperative hemodynamic changes, and use of vasopressor drugs.

## Methods

### Design

This is a prospective follow-up study of two cohorts of hip fracture patients admitted to Ullevål University Hospital or Diakonhjemmet Hospital in Oslo, Norway. Cohort 1 was recruited from September 2005 through December 2006, and Cohort 2 from September 2009 through January 2012. This is an analysis of merged data from these cohorts. The methods of the individual studies have previously been described [1, 22].

### Participants

Patients acutely admitted with a hip fracture (femoral neck, trochanteric or sub-trochanteric) were eligible for inclusion. Patients were excluded if the hip fracture resulted from high-energy trauma or if they were considered as moribund by the orthopaedic surgeon at admission. Patients from nursing homes and patients with dementia were not excluded. Cohort 1 excluded patients < 65 years and patients with a length of hospital stay of < 48 hours.

### Baseline data

Demographic and social information, medical diagnoses, drug use, and information regarding anaesthetic and surgical procedures and complications were collected from medical and anaesthesiologic records, proxy interviews and questionnaires, patient interviews, and information from staff. Proxies provided information regarding pre-fracture cognitive function, using the short form of the Informant Questionnaire on Cognitive Decline in the Elderly (IQCODE-SF) (range 1–5; a score of 5 meaning much worse cognitive function the last 10 years [23], and status of activities of daily living (ADL), using the Barthel ADL Index [24] (range 0–20; a score less than 20 indicates an impairment in basic ADL) and the Nottingham Extended ADL (NEADL) Index (range 0–66; lower scores meaning impairment in complex ADL) [25]. Medical factors included the Charlson Comorbidity Index (CCI) [26], American Society of Anaesthesiologists’ (ASA) score [27], type of fracture, and body mass index (BMI). Clinical findings on admission, during and after the operation included systolic (SBP) and diastolic blood pressure (DBP), heart rate, body temperature and oxygen saturation. Type of surgery performed, type and duration of anaesthesia, and the need for vasopressors or blood transfusion were registered. Duration of surgery and anaesthesia were registered slightly differently in the two cohorts. We therefore categorised the time spent in anaesthesia into quartiles, separately for each cohort.

### Delirium assessments

All patients were screened once daily for delirium using the Confusion Assessment Method (CAM) [28] preoperatively and until the fifth postoperative day (all) or until discharge (delirious patients). The study geriatricians or study nurses performed all assessments. The researchers were trained in delirium assessments and not involved in the care of the patients. The CAM score was based on information from nurses, close relatives and hospital records related to the preceding 24 hours, in combination with a 10 to 30 minute interview with the patient, including tests of cognition, attention and alertness. Delirium severity was measured with the Memorial Delirium Assessment Scale (MDAS), (range of total sum is 0–30, in which 0 means no delirium symptoms) [29]. Patients were assessed regularly on weekdays. Staff members who worked on weekends were interviewed every Monday, and the case notes were scrutinized to identify episodes of delirium. The inter-rater agreement of the delirium diagnosis showed a kappa-value of 1 in both cohorts.

### Procedure for diagnosing dementia at baseline and at follow-up

An experienced geriatrician (TBW) and an experienced specialist in old age psychiatry (KE) independently assessed whether a patient fulfilled the criteria for dementia, at baseline and at follow-up. In case of disagreement, a consensus diagnosis was made. They applied the International Classification of Diseases, version 10 (ICD-10) research criteria for dementia, [30] and used medical records (clinical history, previous diagnosis, test results), cognitive tests and proxy interviews for the diagnosis. The retrospective diagnosis of pre-fracture dementia at baseline was based on information from medical records, IQCODE-SF [23], Barthel ADL Index [24] and NEADL [25] scores, Mini Mental State Examination (MMSE) [31], and The Clock Drawing Test (CDT) [32]. The cognitive test results were only used for dementia diagnosis purposes if the patient did not develop delirium in the acute phase. The following cognitive tests were used at follow-up: MMSE [31] the CDT [32], CERAD 10 word memory task [33] and the Digit Span Task (by WAIS) [34]. Patients recruited during the year 2006 were visited 6 months after surgery, and patients recruited in 2009 through 2012 were visited 12 months after surgery, all at their current place of residence. The assessors were blind to delirium status during hospital stay. We used the consensus diagnoses for the analyses of new-onset dementia developing after the hip fracture. However, for the analyses of incident delirium, patients were considered to have pre-fracture cognitive impairment if their IQCODE-SF average score was 3.44 or higher, as not all cases in Cohort 1 had an expert-based dementia diagnosis. The IQCODE-SF is well validated for this purpose [35,36].

### Measurement of blood pressure

Vital signs, including non-invasive blood pressure, were recorded during anaesthesia according to hospital routines. Intraoperative blood pressure management was left to the discretion of the caring anaesthesiologist. Data was extracted from the perioperative anaesthesia chart (both electronic and paper records), and the lowest documented blood pressure during anaesthesia was used for analyses. Artifacts were excluded based on a clinical judgement. Preoperative measurements were recorded at admission, and postoperative measurements were recorded once 4 hours after surgery. Mean arterial pressure (MAP) was calculated as [(SBP + (2 x DBP))/3). Blood pressure was analysed as a continuous variable, but relevant thresholds were also explored: a MAP < 60 mm Hg, a MAP < 50 mm Hg, a MAP decrease > 30% and a MAP decrease > 40% relative to admission MAP.

### Follow-up

The researchers doing the follow-up visits were blinded to all clinical data including delirium status during the acute hospital stay. The follow-up visits included comprehensive cognitive testing, and an interview with proxies regarding cognitive and physical (ADL) function, using the same questionnaires as during the hospital stay. In Cohort 1, follow-up visits were performed by one researcher. In Cohort 2 they were done by one of two research nurses.

### Statistical analyses

The sample size was driven by convenience. We performed three sets of analyses: one for preoperative delirium, one for incident delirium and one for new-onset dementia at follow-up. Potential risk factors were selected based on literature and expert opinion. As dementia is a strong risk factor for delirium, we stratified the analyses for incident delirium according to pre-fracture cognitive status, to see if potential risk factors differed between the two strata. We used Student’s t-tests (normally distributed data) or Mann-Whitney U-tests (skewed data) to compare continuous variables. Categorical variables were analysed using Chi-square tests.

Variables with p-values ≤ 0.10 in univariate analyses were chosen for the multivariate logistic regression models. We followed a stepwise procedure for variable selection. First, models for background data, admission data and perioperative data, respectively, were developed. We decided to explore the potential risk factors in each of these categories separately in the intermediate models, and then to merge the significant variables (p-values < 0.05) from each of these categories in the final multivariate model. Both forward and backward stepping procedures for variable selection were used, though they yielded the same models. Continuous variables were categorised into quartiles or quintiles to check for linearity. If their relationship to the outcome was not homogenous through their entire range, they were recoded into ordinal variables (cutpoints based on quartiles or quintiles) or dichotomised as appropriate based on the preliminary analyses. We assessed for confounders, and investigated multicollinearity using a correlation matrix and by exploring the variance inflation factor (VIF). If there was correlation above 0.6, one of the variables was omitted. Statistical analyses were performed using SPSS Statistics version 21 (IBM, Armonk NY).

### Ethical considerations

Ethical approval for this study was provided by the Regional Committee for Ethics in Medical Research in Norway. Cohort 1 (REK s-05075) was approved on 18 April 2005 (Chairperson Prof. K.Engedal) and Cohort 2 (REK S-09169a) on 25 March 2009 (Chairperson Prof. G. Nicolaysen). Both trials were also approved by the Data Protection Authorities and undertaken in accordance with the Declaration of Helsinki. Patients were enrolled based either on written consent or on presumed consent in combination with written assent from the nearest relative.

## Results

**Fig 1:**
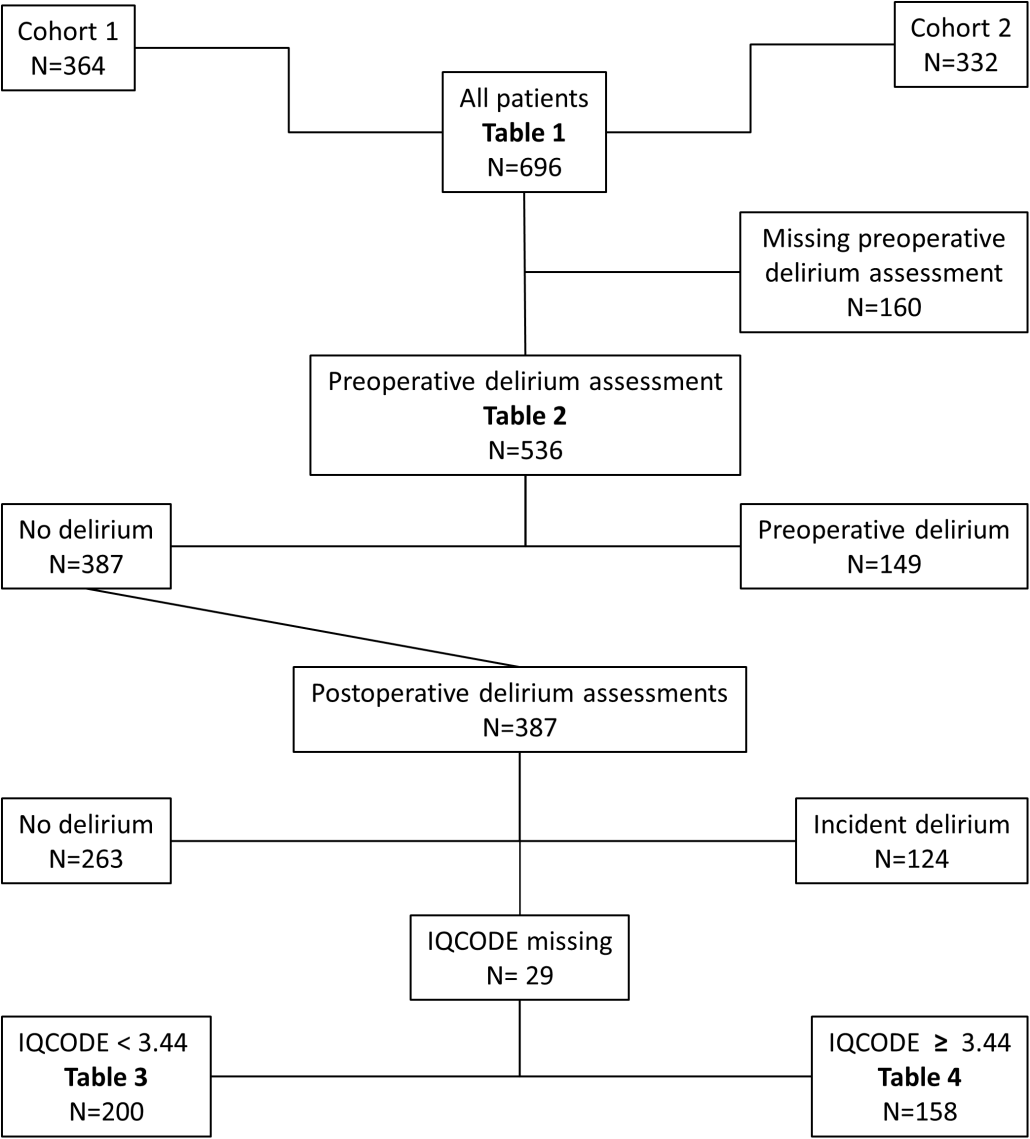
Study flowchart. Delirium assessments.

In all, 696 patients were included in the study, 364 from Cohort 1 and 332 from Cohort 2 (Fig 1). Of the included patients, 536 were assessed for delirium preoperatively; 149 (28%) had preoperative delirium, whereas 387 patients had no preoperative delirium and were eligible for analysis of incident delirium. Preoperative delirium assessments are missing for 127 patients in cohort 1 and 33 patients in cohort 2 because the assessors (study nurses/physician) did not manage to see the patients before the surgery. Characteristics of the two cohorts are shown in Table 1. Sepsis was registered in 9/696 patients (1.3%) and stroke in 4/696 patients (0.6%). There were no significant differences in the incidences between the two cohorts. In the group with IQCODE<3.44, 2/34 with incident delirium had sepsis, versus 0/166 in the non-delirious group, p = 0.03. The numbers were too small to perform further reasonable statistical analyses. No hemodynamic data were excluded because of artifacts.

**Table 1:** Descriptive data of the two cohorts.

	Cohort 1	Cohort 2
	N = 364	N = 332
**Characteristic**		
**Background data**		
Age, median (IQR)	84 (79–88)	85 (78–89)
Female, n/N (%)	276/364 (76)	252/332 (76)
Prefracture Barthel Index score, median (IQR)	19 (16–20)	18 (14–20)
Nottingham EADL, median (IQR)	39 (17–56)	30(10–52)
Prefracture dementia (consensus diagnosis), n/N (%)	65/171 (38)	165/332 (50)
IQCODE score, median (IQR)	3.44 (3.06–4.50)	3.75 (3.06–4.81)
Living in an institution, n/N (%)	56/364 (15)	104/332 (31)
Injury occurred indoors, n/N (%)	259/361 (72)	277/332 (83)
Prefracture hypertension, n/N (%)	121/364 (33)	136/315 (43)
Charlson comorbidity index > 1, n/N (%)	107/364 (29)	108/332 (33)
Polypharmacy (= 5 or more systemic medications), n/N (%)	140/364 (39)	159/332 (48)
BMI, median (IQR)^b^	22 (20–25)	24 (21–27)
**Admission data**		
Time admission to surgery, hours, median (IQR)	18 (9–29)	24 (16–40)
CRP > 10 mg/L, n/N (%)	129/364 (35)	130/328 (40)
ASA group III, IV or V, n/N (%)	181/363 (50)	191/306 (62)
Spinal anaesthesia, n/N (%)	337/362 (93)	291/315 (92)
Surgery started between 5:00 p.m. and 6:30 a.m.	153/360 (43)	156/328 (48)
Sepsis, n/N (%)	5/364 (1)	4/332 (1)
Stroke, n/N (%)	3/364 (1)	1/332 (0)
Delirium any time during hospital stay	168/364 (46)	168/327 (51)
Preoperative delirium	50/237 (21)	99/299 (33)
Incident delirium	68/187 (36)	56/200 (26)

IQR = Interquartile range; Nottingham EADL = Nottingham Extended Activities of Daily Living; IQCODE = Informant Questionnaire on Cognitive Decline in the Elderly; BMI = Body Mass Index; CRP = C-reactive protein; ASA = American Society of Anaesthesiologists

Missing data (N) for several variables: Prefracture dementia consensus diagnosis: N = 193, Injury occurred indoors: N = 3, Prefracture hypertension: N = 17, BMI: N = 134, CRP: N = 4, ASA group: N = 27, Spinal anaesthesia: N = 19, Time of surgery: N = 8, Delirium any time: N = 5, Preoperative delirium assessments: N = 160 (127 in cohort 1 and 33 in cohort 2).

### Preoperative delirium

Patients with preoperative delirium were significantly older and more functionally impaired, more often had preoperative cognitive impairment, were more often living in an institution and were more likely to have fallen indoors (Table 2). They presented with more polypharmacy, had higher heart rate and lower SpO_2_, were in higher ASA groups and had a longer waiting time from admission to surgery than those without delirium.

**Table 2:** Risk factors for preoperative delirium, n = 536^a^.

	No delirium	Delirium	
**Characteristic**	N = 387 (72%)	N = 149 (28%)	P-value^b^
**Background data**			
Age, median (IQR)	84 (77–88)	85 (80–89)	0.02
Female, n/N (%)	298/387 (77)	107/149 (72)	0.22
IQCODE ≥ 3.44, n/N (%)	158/358 (44)	115/136 (85)	<0.001
Living in an institution, n/N (%)	61/387 (16)	68/149 (46)	<0.001
Injury occurred indoors, n/N (%)	276/385 (72)	136/149 (91)	<0.001
Barthel Index 19 or 20, n/N (%)	209/362 (58)	29/137 (21)	<0.001
Prefracture hypertension, n/N (%)	159/374 (43)	51/145 (35)	0.14
Charlson comorbidity index > 1, n/N (%)	116/387 (30)	53/149 (36)	0.22
Polypharmacy (= 5 or more medications), n/N (%)	157/387 (41)	80/149 (54)	0.01
BMI < 20.0 kg/m2, n/N (%)	65/339 (19)	22/105 (21)	0.68
Time admission to surgery, hours, median (IQR)	22 (15–35)	36 (23–50)	<0.001
**Admission data**			
CRP > 10 mg/L, n/N (%)	139/385 (36)	65/147 (44)	0.09
Body temp 37.5 C or more, n/N (%)	113/382 (30)	54/146 (37)	0.12
ASA group III, IV or V, n/N (%)	188/371 (51)	101/140 (72)	<0.001
MAP at admission, mmHg, median (IQR)	107 (95–118)	106 (95–118)	0.66
HR at admission, bpm, median (IQR)	80 (70–92)	84 (70–96)	0.01
SpO_2_% at admission, median (IQR)	95 (93–97)	95 (92–97)	0.04

IQR = Interquartile range; IQCODE = Informant Questionnaire in Cognitive Decline in the Elderly; BMI = Body Mass Index; CRP = C-reactive protein; ASA = American Society of Anaesthesiologists; MAP = Mean Arterial Pressure; Heart Rate; bpm = beats per minute

Missing data (N) for several variables: IQCODE: N = 42, Injury occurred indoors: N = 2, Barthel Index: N = 37, Prefracture hypertension: N = 17, BMI: N = 92, CRP: N = 4, Body temp: N = 8, ASA group: N = 25, Spinal anaesthesia: N = 19, Time of surgery: N = 8, Delirium any time: N = 5, Preoperative delirium assessments: N = 160 (127 in cohort 1 and 33 in cohort 2).

^a^ Preoperative delirium assessments are missing in 160 patients

^b^ Mann-Whitney tests for skewed continuous variables, chi-square tests for categorical variables, p-values are 2-tailed.

Preoperative delirium data are missing for 127 patients in cohort 1 and 33 patients in cohort 2 (Table 3). The reason for the missing data is that the assessors (study nurses/physician) did not manage to assess the patients before surgery. The prevalence of prefracture dementia and other baseline characteristics were equal between these groups.

**Table 3:** Patients missing preoperative delirium assessments.

	**No preoperative delirium assessment**	**All others**	**p-value**^a^
	N = 160	N = 536	
BMI, median (IQR)	22 (19–24)	23 (21–26)	0.01
Time admission to surgery, hours, median (IQR)	10 (5–20)	24 (16–41)	<0.001
Surgery started between 5:00 p.m. and 6:30 a.m., n/N (%)	90/160 (56)	219/528 (42)	0.001

BMI = Body Mass Index; IQR = Interquartile range. Missing data: BMI: N = 134, Time of surgery, N = 8

^a^ Mann-Whitney tests for skewed continuous variables, chi-square tests for categorical variables, p-values are 2-tailed.

### Incident delirium

Of the 387 patients who were free from delirium preoperatively, 124 (32%) developed delirium after surgery (incident delirium). In 29 of the 387 patients, an ICQODE score was missing. They were excluded from further analyses (Fig 1). Among patients with an IQCODE score < 3.44 (i.e. no preoperative cognitive impairment; Table 4), univariate analyses showed that patients with delirium were more likely to have an NEADL score of < 45 points and a BMI of < 20 kg/m^2^, to be in ASA group III or higher, or to receive ≥ 2 blood transfusions. In the final multivariate model, having a BMI of < 20 kg/m^2^, having an NEADL score of < 45 points, being in ASA group ≥ 3, and receiving ≥ 2 blood transfusions were found to be independently associated with incident delirium.

**Table 4:** Risk factors for incident delirium in patients with IQCODE <3.44, n = 200.

Characteristic	No incident delirium	Incident delirium	Univariate models			Intermediate multivariate models		Final multivariate model		
	N = 166 (83%)	N = 34 (17%)	OR	95% CI	P-value^a^	OR	95% CI	OR	95% CI	P-value^a^
**Background data**										
Age > 82 years, n/N (%)	75/166 (45)	21/34 (62)	2.0	0.9–4.2	0.08					
NEADL < 45 points, n/N (%)	33/162 (20)	14/33 (42)	2.9	1.3–6.3	0.010	2.9	1.2–6.9	2.6	1.0–6.7	0.045
BMI < 20.0 kg/m2, n/N (%)	20/145 (14)	13/33 (39)	4.1	1.7–9.4	0.002	4.5	1.8–11.1	6.5	2.3–18.7	0.001
Time admission to surgery > 24 hours, n/N (%)	77/164 (47)	11/33 (33)	0.6	0.3–1.2	0.15					
**Admission and preoperative data**										
ASA group III, IV or V, n/N (%)	54/158 (43)	21/32 (66)	3.7	1.7–8.2	0.001	3.5	1.6–7.8	3.0	1.2–7.9	0.023
CRP > 10 mg/L, n/N (%)	50/165 (30)	16/34 (47)	2.0	1.0–4.3	0.07					
MAP at admission, mmHg, median (IQR)	106 (97–115)	111 (100–123)			0.18					
HR at admission, bpm, median (IQR)	81 (69–92)	82 (68–93)			0.34					
**Per- and postoperative data**										
Type of anaesthesia										
General anaesthesia	15/163 (9)	2/33 (6)	0.6	0.1–2.9	0.56					
Spinal or epidural anaesthesia	148/163 (91)	31/33 (94)								
Duration of anaesthesia, in quartiles					0.74					
Q1	39/160 (24)	6/33 (18)								
Q2	45/160 (28)	8/33 (24)	1.2							
Q3	39/160 (24)	9/33 (27)	1.5							
Q4	37/160 (23)	10/33 (30)	1.8							
Being in Q4 vs Q1-Q3			1.4	0.6–3.3	0.38					
Received benzodiazepine iv perioperatively, n/N (%)	72/166 (43)	12/34 (35)			0.45					
Lowest MAP during anaesthesia, mmHg, median (IQR)	67 (57–78)	63 (55–72)			0.24					
Difference in MAP from admission to lowest value during anaesthesia					0.44					
≤ 30 mmHg, n/N (%)	58/162 (36)	7/33 (21)								
31–40 mmHg, n/N (%)	27/162 (17)	6/33 (18)	1.8							
41–50 mmHg, n/N (%)	25/162 (15)	6/33 (18)	2.0							
> 50 mmHg, n/N (%)	52/162 (32)	14/33 (42)	2.2							
Pressor used during surgery, n/N (%)	72/164 (44)	20/34 (59)	1.8	0.9–3.9	0.13					
MAP postoperatively, mmHg, median (IQR)	83 (75–95)	81 (71–87)			0.43					
HR postoperatively, beats per minute					0.51					
< 66 bpm, n/N (%)	33/162 (20)	5/32 (16)								
66–75 bpm, n/N (%)	39/162 (24)	7/32 (22)	1.2							
76–85 bpm, n/N (%)	48/162 (30)	8/32 (25)	1.1							
86–95 bpm, n/N (%)	24/162 (15)	9/32 (28)	2.5							
> 95 bpm, n/N (%)	18/162 (11)	3/32 (9)	1.1							
SpO_2_% postoperatively, median (IQR)	96 (95–98)	96 (93–97)			0.061	0.8	0.7–1.0			
Received ≥ 2 blood transfusions (SAG units), n/N (%)	46/161 (29)	20/34 (59)	3.6	1.7–7.7	0.001	3.4	1.5–7.6	3.2	1.3–7.8	0.012

The intermediate multivariate models consist of variables from the same main category (Background, Admission, Per- and Postoperative), and variables that turned out to be significant in these analyses were candidates for the final model.

IQCODE = Informant Questionnaire in Cognitive Decline in the Elderly; OR = Odds Ratio; CI = Confidence Interval; NEADL = Nottingham Extended Activities of Daily Living; BMI = Body Mass Index; ASA = American Society of Anaesthesiologists; CRP = C -reactive protein; MAP = Mean Arterial Pressure; IQR = Interquartile range; HR = Heart Rate; bpm = beats per minute

Missing data (N) for several variables: NEADL: N = 5, BMI: N = 22, Time to surgery: N = 3, ASA group: N = 10, CRP: N = 1, Type of anaesthesia: N = 4, Duration of anaesthesia: N = 7, Difference in MAP from admission to lowest value during anaesthesia: N = 5, Pressor used during surgery: N = 2, HR postoperatively: N = 6, Blood transfusions: N = 5.

^a^ Mann-Whitney tests for skewed continuous variables, p-values are 2-tailed. Logistic regression for categorical and ordinal variables.

In patients with a preoperative IQCODE score of ≥ 3.44 (i.e. cognitive impairment), those who developed incident delirium more often received benzodiazepine perioperatively (Table 5).

**Table 5:** Risk factors for incident delirium in patients with IQCODE ≥3.44, n = 158.

Characteristic	No incident delirium	Incident delirium	Univariate models			Intermediate multivariate models		Final multivariate models		
	N = 81 (51%)	N = 77 (49%)	OR	95% CI	P-value^a^	OR	95% CI	OR	95% CI	p-value^a^
**Background data**										
Age > 82 years, n/N (%)	48/81 (59)	62/77 (81)	2.8	1.4–5.8	0.005	2.9	1.4–6.2	3.2	1.5–6.8	0.003
NEADL < 45 points, n/N (%)	61/81(75)	67/76 (88)	2.4	1.0–5.8	0.042	2.3	0.9–5.6	2.6	1.0–6.5	0.04
BMI < 20.0 kg/m2, n/N (%)	11/70 (16)	16/67 (24)	1.7	0.7–4.0	0.284					
Time admission to surgery > 24 hours, n/N (%)	28/79 (35)	22/77 (29)	0.7	0.4–1.4	0.394					
**Admission and preoperative data**										
ASA group III, IV or V, n/N (%)	46/78 (59)	47/74 (64)	1.2	0.6–2.3	0.619					
CRP > 10 mg/L, n/N (%)	38/81 (47)	26/76 (34)	0.6	0.3–1.1	0.143					
MAP at admission, mmHg, median (IQR)	105 (92–119)	105 (92–116)			0.718					
HR at admission, bpm, median (IQR)	80 (70–89)	78 (68–96)			0.720					
**Per- and postoperative data**										
Type of anaesthesia					0.207					
General anaesthesia	1/78 (1)	4/76 (5)								
Spinal or epidural anaesthesia	77/78 (99)	72/76 (95)								
Duration of anaesthesia, in quartiles					0.878					
Q1	21/77 (27)	18 /77 (23)								
Q2	20/77 (26)	19/77 (25)	1.1							
Q3	19/77 (25)	19/77 (25)	1.2							
Q4	17/77 (22)	21/77 (27)	1.4							
Being in Q4 vs Q1- Q3	17/77 (22)	21/77 (27)	1.3	0.6–2.8	0.575					
Received benzodiazepine iv perioperatively, n/N (%)	23/81 (28)	38/77 (50)	2.5	1.3–4.7	0.009	2.5	1.3–4.7	2.8	1.4–5.7	0.004
Lowest MAP during anaesthesia, mmHg, median (IQR)	65 (55–82)	67 (62–75)			0.450					
Difference in MAP from admission to lowest value during anaesthesia					0.191					
≤ 30 mmHg, n/N (%)	35/79 (44)	29/76 (38)								
31–40 mmHg, n/N (%)	9/79 (11)	18/76 (24)	2.4							
41–50 mmHg, n/N (%)	11/79 (14)	12/76 (16)	1.3							
> 50 mmHg, n/N (%)	24/79 (30)	17/76 (22)	0.9							
Pressor used during surgery, n/N (%)	41/80 (51)	30/77 (39)	0.6	0.3–1.1	0.149					
MAP postoperatively, mmHg, median (IQR)	90 (75–96)	89 (78–93)			0.915					
HR postoperatively, beats per minute					0.609					
< 66 bpm, n/N (%)	14/80 (18)	10/76 (13)								
66–75 bpm, n/N (%)	18/80 (23)	21/76 (28)								
76–85 bpm, n/N (%)	17/80 (21)	14/76 (18)								
86–95 bpm, n/N (%)	13/80 (16)	18/76 (24)								
> 95 bpm, n/N (%)	18/80 (23)	13/76 (17)								
SpO_2_% postoperatively, median (IQR)	96 (94–98)	97 (95–98)			0.246					
Recevied blood transfusion during stay, n/N (%)	38/81 (47)	40/77 (52)	0.8	0.4–1.5	0.633					
Received ≥ 2 blood transfusions (SAG units), n/N (%)	35/81 (43)	32/77 (42)	0.9	0.5–1.8	0.873					

The intermediate multivariate models consist of variables from the same main category (Background, Admission, Per- and Postoperative), and variables that turned out to be significant in these analyses were candidates for the final model.

IQCODE = Informant Questionnaire in Cognitive Decline in the Elderly; IQR = Interquartile range; NEADL = Nottingham Extended Activities of Daily Living; BMI = Body Mass Index; ASA = American Society of Anaesthesiologists; CRP = C -reactive protein; MAP = Mean Arterial Pressure; HR = Heart Rate; bpm = beats per minute

Missing data (N) for several variables: NEADL: N = 1, BMI: N = 21, Time to surgery: N = 2, ASA group: N = 6, CRP: N = 1, Type of anaesthesia: N = 4, Duration of anaesthesia: N = 4, Difference in MAP from admission to lowest value during anaesthesia: N = 3, Pressor used during surgery: N = 1, HR postoperatively: N = 2.

^a^ Mann-Whitney tests for skewed continuous variables, p-values are 2-tailed. Logistic regression for categorical and ordinal variables.

No significant associations were found between incident delirium and perioperative haemodynamic variables, type or duration of anaesthesia, or the administration of a vasopressor, neither in those with nor without preoperative cognitive impairment.

### New onset dementia

**Fig 2:**
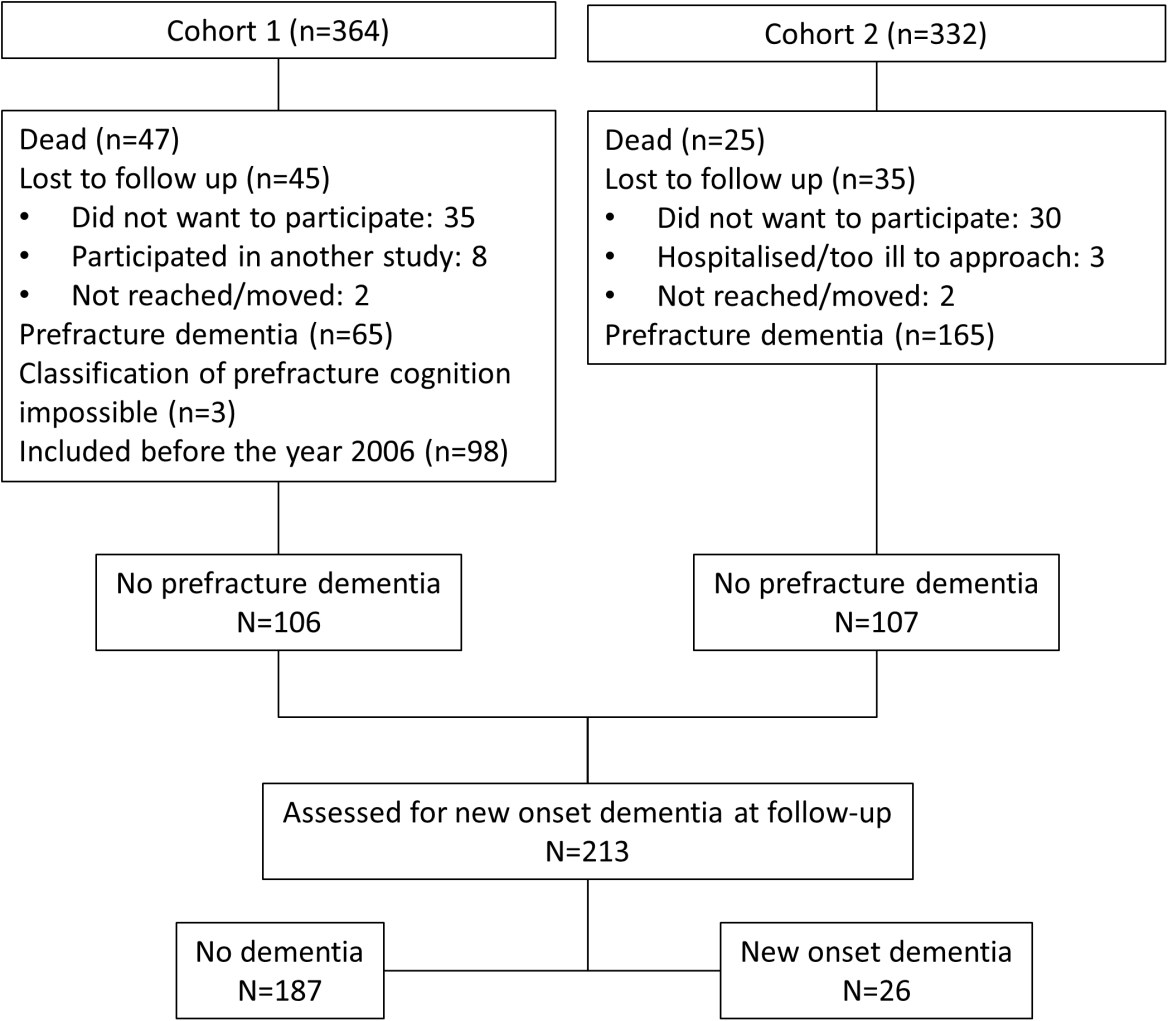
Study flowchart. Assessment of new onset dementia.

Of all the patients, 273 were free from pre-fracture dementia, of which 213 were assessed at six (n = 106) or 12 (n = 107) months postoperatively, and 26 (12%) were diagnosed with incident dementia (Fig 2). In univariate analysis, these patients were significantly older, had lower NEADL scores, were in higher ASA groups, and more often had delirium during their index stay than patients without new-onset dementia (Table 6). Furthermore, they had significantly higher MAP at admission, as well as postoperatively, and they more often received vasopressor drugs during surgery. They tended to have a greater fall in MAP from admission to the peroperative state, but this difference was not statistically significant.

**Table 6:** Risk factors for new onset dementia, n = 213.

Characteristic	No dementia	New onset dementia	Univariate models			Intermediate multivariate models		Final multivariate model		
	N = 187 (88%)	N = 26 (12%)	OR	95% CI	P-value^a^	OR	95% CI	OR	95% CI	P-value^a^
**Background data**										
Age > 82 years, n/N (%)	87/187 (47)	23/26 (89)	8.8	2.6–30.4	<0.001	8.7	1.9–39.2	8.8	1.9–41.5	0.006
Prefracture NEADL < 45 points, n/N (%)	46/166 (28)	11/20 (55)	3.2	1.2–8.2	0.016	2.7	1.0–7.1			
Prefracture Barthel Index 19 or 20, n/N (%)	134/173 (78)	14/20 (70)	0.7	0.2–1.9	0.457					
Prefracture hypertension, n/N (%)	73/179 (41)	12/25 (48)	1.3	0.6–3.1	0.494					
Charlson comorbidity index >1, n/N (%)	47/187 (25)	10/26 (39)	1.9	0.8–4.4	0.155					
Polypharmacy (= 5 or more medic), n/N (%)	59/187 (32)	13/26 (50)	2.2	0.9–5.0	0.077					
Injury occurred indoors, n/N (%)	118/187 (63)	21/26 (81)	0.4	0.2–1.1	0.083					
**Admission data**										
ASA group III, IV or V, n/N (%)	70/180 (39)	16/25 (64)	2.8	1.2–6.7	0.029	2.6	1.0–6.4	3.5	1.1–11.4	0.038
MAP at admission					0.022					
< 110 mmHg, n/N (%)	105/187 (56)	8/26 (31)					P = 0.035			
111–119 mmHg, n/N (%)	31/187 (17)	4/26 (15)	1.7			1.9				
120–129 mmHg, n/N (%)	32/187 (17)	6/26 (23)	2.5			3.0				
≥ 130 mmHg, n/N (%)	19/187 (10)	8/26 (31)	5.5			5.3				
HR at admission, bpm, median (IQR)	80 (70–90)	80 (70–103)			0.274					
**Per- and postoperative data**										
Type of anaesthesia										
General anaesthesia	14/181 (8)	2/26 (8)	1.0	0.2–4.6	0.99					
Spinal or epidural anaesthesia	167/181 (92)	24/26 (92)								
Duration of anaesthesia, in quartiles					0.40					
Q1	42/175 (24)	6/25 (24)								
Q2	51/175 (29)	4/25 (16)								
Q3	43/175 (25)	6/25 (24)								
Q4	39/175 (22)	9/25 (36)								
Being in Q4 vs Q1-Q3	39/175 (22)	9/25 (36)			0.14					
Received benzodiazepine iv perioperatively, n/N (%)	68/187 (36)	12/26 (46)			0.39					
Difference in MAP from admission to lowest value during anaesthesia	39 (24–54)	50 (41–61)			0.068					
≤ 30 mmHg, n/N (%)	62/184 (34)	3/25 (12)								
31–40 mmHg, n/N (%)	37/184 (20)	3/25 (12)	1.7							
41–50 mmHg, n/N (%)	27/184 (15)	6/25 (24)	4.6							
> 50 mmHg, n/N (%)	58/184 (32)	13/25 (52)	4.6							
Pressor used during surgery, n/N (%)	79/185 (43)	19/26(73)	3.6	1.4–9.1	0.006	4.8	1.8–12.8	3.5	1.2–10.7	0.027
MAP postoperatively					0.027					
≤ 80 mmHg, n/N (%)	71/184 (39)	6/26 (23)					P = 0.009			0.027
81–90 mmHg, n/N (%)	53/184 (29)	5/26 (19)	1.1			1.4		1.5	0.3–6.6	
91–100 mmHg, n/N (%)	38/184 (21)	6/26 (23)	1.9			2.0		4.0	0.9–17.7	
> 100 mmHg, n/N (%)	22/184 (12)	9/26 (35)	4.8			7.3		10.6	2.1–55.3	
HR postoperatively, bpm					0.082					
< 66 bpm, n/N (%)	42/184 (23)	2/26 (8)								
66–75 bpm, n/N (%)	41/184 (22)	2/26 (8)	1.0							
76–85 bpm, n/N (%)	44/184 (24)	12/26 (46)	5.7							
86–95 bpm, n/N (%)	31/184 (17)	6/26 (23)	4.1							
> 95 bpm, n/N (%)	26/184 (14)	4/26 (15)	3.2							
Received ≥2 blood transfusions (SAG units), n/N (%)	66/184 (36)	10/26 (39)			0.83					
SpO_2_% postoperatively, median (IQR)	96 (95–98)	96 (94–98)			0.191					
Delirium										
Delirium (anytime) during hospital stay, n/N (%)	38/187 (20)	17/26 (65)	7.4	3.1–17.9	< 0.001	7.4	3.1–17.9	6.7	2.3–20.1	0.001

The intermediate multivariate models consist of variables from the same main category (Background, Admission, Per- and postoperative, Delirium), and variables that turned out to be significant in these analyses were candidates for the final model.

OR = Odds Ratio; CI = Confidence Interval; NEADL = Nottingham Extended Activities of Daily Living; ASA = American Society of Anaesthesiologists; MAP = Mean Arterial Pressure; HR = Heart Rate; bpm = beats per minute; IQR = Interquartile range.

Missing data (N) for several variables: NEADL: N = 21, Barthel Index: N = 20, Prefracture hypertension: N = 9, ASA group: N = 8, Type of anaesthesia: N = 6, Duration of anaesthesia: N = 13, Difference in MAP from admission to lowest value during anaesthesia: N = 4, Pressor used during surgery: N = 2, MAP and HR postoperatively: N = 2, Blood transfusions: N = 3.

^a^ Mann-Whitney tests for skewed continuous variables, p-values are 2-tailed.Logistic regression for categorical and ordinal variables.

In the final multivariate model, being more than 82 years old, being in ASA group ≥ 3, having received a vasopressor during surgery, having higher MAP postoperatively and having suffered from delirium during hospital stay were found to be independently associated with new-onset dementia at follow-up.

The diagnosis of hypertension was forced into the models for both incident delirium and new onset dementia, to account for the possibility that chronic hypertension may affect the consequence of transient hypotension; this did not modify the results. Sensitivity analyses using continuous variables did not change the final models significantly.

In Cohort 1, 92 patients were lost to follow up (Fig 2). These patients did not differ from the 174 patients included with regard to gender, age, delirium or prefracture cognitive function. The statistically significant differences between the patients lost to follow-up at 12 months in cohort 2 are presented in Table 7:

**Table 7:** Cohort 2—patients lost to follow up at 12 months in the original sample, n = 332.

**Characteristic**	**Assessed at 12 months**	**Lost to follow-up at 12 months**	**P-value**^a^
	N = 193	N = 139	
Prefracture dementia, n/N (%)	87/193 (45)	78/139 (56)	0.06
Delirium any time, n/N (%)	85/192 (44)	83/135 (62)	0.002
BMI, median (IQR)	22 (24–28)	21 (23–27)	0.03
Barthel index, median (IQR)	18 (15–20)	17 (12–19)	0.02
NEADL, median (IQR)	36(12–54)	24 (8–49)	0.02
MAP at admittance, median (IQR)	107(93–119)	104 (87–15)	0.04
MAP postop, median (IQR)	84(75–94)	80 (72–89)	0.01

BMI = Body Mass Index; IQR = Interquartile range; NEADL = Nottingham Extended Activities of Daily Living; MAP = Mean Arterial Pressure.

Missing data: Delirium any time: N = 5

^a^ Mann-Whitney tests; Chi-square tests; p-values are 2-tailed.

## Discussion

The three main findings in this study of hip fracture patients are:

First, postoperative hypertension and the use of vasopressors during surgery are both significantly associated with new-onset dementia at follow-up.

Hypertension is a well-known risk factor for cognitive impairment and dementia [37]. Patients with new-onset dementia in the current study had significantly higher MAP at admission and postoperatively than those without dementia at follow-up. The difference remained significant for postoperative MAP in the final adjusted model, also after adjusting for a previous diagnosis of hypertension. Our finding may be a result of undiagnosed pre-fracture hypertension. There was a tendency, although not statistically significant, for patients with new-onset dementia to have a more pronounced reduction in MAP during anaesthesia. One reason might be that chronic hypertension, with higher sympathetic tone, stiffer blood vessels and altered haemodynamic regulation, makes the cardiovascular system more susceptible to the effects of vasodilatation and sympatholysis caused by anaesthesia [38]. Higher postoperative blood pressure values could also be a result of the correction of intraoperative hypotension, either with vasopressors or blood transfusions. We did not find that high blood pressure values were associated with delirium, indicating that the impact of hypertension on cognitive functioning is more chronic than acute.

It could also be that patients with a subclinical neurodegenerative disease have a dysregulation of blood pressure, making them more prone to perioperative hypotension [39]. The individual autoregulatory MAP threshold for adequate brain oxygen supply varies among patients, and MAP values well tolerated in some patients may be inadequate in others [40].

The association between perioperative use of adrenergic agonists and new-onset dementia was statistically significant, even after adjusting for perioperative decrease in MAP, suggesting that the use of vasopressors might not only indicate relative or absolute hypotension. The same trend was present, although not statistically significant, among patients free from pre-fracture cognitive impairment who developed delirium postoperatively. A causative role of vasopressor use on new onset dementia is absolutely unclear and maybe unlikely. However, this finding is biologically interesting. Low cerebral tissue oxygen saturation (SctO_2_) might be associated with poor postoperative cognitive outcomes [21]. Phenylephrine bolus treatment is found to decrease cerebral tissue oxygen saturation (SctO_2_), even when MAP increases [20]. Elderly brains are probably more vulnerable to ischemia, as many have pre-existing neurodegenerative or neurovascular pathology. Thus, they may be more prone to adverse effects of transient falls in SctO_2_ than otherwise healthy younger patients. Our finding needs to be confirmed in future studies, as this retrospective study was not primarily designed for exploring this relationship.

Second, among patients without pre-fracture cognitive impairment, low BMI, high ASA group, low extended ADL function, and receipt of ≥ 2 blood transfusions were independent and significant risk factors for incident delirium. These risk factors were not found in patients with pre-fracture cognitive impairment.

The study suggests different predisposing factors for postoperative delirium in patients with and without pre-fracture cognitive impairment. The vulnerability of a person with cognitive impairment may render most other risk factors for delirium insignificant. Benzodiazepines are known to be associated with an increased risk of delirium [41,42]. Our findings suggest this risk to be particularly relevant in patients with pre-fracture cognitive impairment. Patients with larger cognitive reserves seem to need stronger physiological aberrations, such as major blood loss, to develop postoperative delirium.

Our findings are in line with an earlier study that identified risk factors for postoperative delirium in hip fracture patients and stratified their analyses according to pre-fracture dementia (diagnosis based on preoperative MMSE scores and a clinical diagnosis by a geriatrician) [43].In the group without dementia, the physical illness severity (e.g. ASA classification), a low BMI and the need for transfusion of more than 2 units of red blood cells were significant risk factors for incident delirium [43]. That study, however, did not look at the risk for new onset dementia in the postoperative period.

Third, we found no association between perioperative haemodynamic variables and incident delirium.

One hypothesis in delirium pathophysiology is that intraoperative hypotension, leading to inadequate cerebral perfusion, increases the risk of postoperative delirium [10,44,45]. However, this hypothesis remains controversial, and the data are contradictory. An early study of 111 hip fracture patients found delirium in 92% of the patients who had a severe perioperative drop in BP [10]. Edlund et al. found perioperative hypotension to be an independent risk factor for incident delirium in a similar patient cohort [11]. A recent study of 103 hip fracture patients reported that severe hyper- or hypotension during anaesthesia was associated with delirium on postoperative day 2 [18], but patients with preoperative delirium or severe cognitive impairment were excluded in that study, and delirium was only assessed once. Other studies of delirium and intraoperative hypotension have been carried out in patients undergoing elective surgery [14,15], and several of them excluded patients with preoperative cognitive decline [12,13,16]. Yet another study supports our findings [40]. MAP above the upper limit of autoregulation during cardiopulmonary bypass, and not hypotension, was a risk factor for postoperative delirium in that study.

Strengths of this study are that it comprises two fairly large and similar cohorts of hip fracture patients, including nursing home residents and persons with pre-existing dementia, who otherwise often are excluded. The cognitive assessments, including the dementia evaluations and bedside delirium diagnostics, were conducted using validated and robust methods. Follow-up allowed us to study long term cognitive outcomes.

There are limitations that should be addressed. Although all patients in the current study were included in studies with prospective design, the measurements of perioperative hemodynamic variables were not standardised between the two studies, and some hemodynamic data had therefore to be collected retrospectively. We only registered one blood pressure and heart rate measurement pre- and postoperatively. We did not register the highest blood pressure values peroperatively or the duration of hypotensive episodes. We did not have data related to possible ICU admissions during follow-up. The sample size was driven by convenience. Even if the cohort is large and representative for hip fracture patients admitted to our hospitals, the number of patients with new-onset dementia is small, reducing statistical power for analyses of new-onset dementia and leaving the confidence intervals wide. The sample size was not large enough to include all the candidate variables into the model, and we had to follow a stepwise procedure. Unfortunately, the data are insufficient to properly assess the role of death as a competing risk in our model of new onset dementia, and there were patients lost to follow-up. Another weakness is that there was no delirium assessments on weekends, and that preoperative delirium data are missing for 160 patients. Neither delirium sub-types nor the specific timing of delirium episodes were explored. Time to follow-up was different in the two cohorts. If all patients had been assessed for new-onset dementia at 12 months, the sensitivity of our findings would have been higher.

## Conclusion

In hip fracture patients, delirium, the use of vasopressors during surgery and/or postoperative hypertension is associated with new-onset dementia after the fracture. The possible negative impact of vasopressors on clinical outcomes should be further explored in prospective studies. Risk factors for incident delirium seem to differ according to pre-fracture cognitive status. Low BMI, severity of physical illness, low extended ADL functioning and great blood loss seem to be risk factors for incident delirium in patients without pre-fracture cognitive impairment.
